# German Anxiety Barometer—Clinical and Everyday-Life Anxieties in the General Population

**DOI:** 10.3389/fpsyg.2016.01344

**Published:** 2016-09-09

**Authors:** Dirk Adolph, Silvia Schneider, Jürgen Margraf

**Affiliations:** ^1^Department of Clinical Psychology and Psychotherapy, Faculty of Psychology, Mental Health Research and Treatment Center, Ruhr Universität BochumBochum, Germany; ^2^Department of Clinical Child and Adolescent Psychology, Faculty of Psychology, Mental Health Research and Treatment Center, Ruhr University BochumBochum, Germany

**Keywords:** anxiety disorders, anxiety across the lifespan, everyday-life anxieties, epidemiological data, representative data

## Abstract

The objective of this study was to test a time-efficient screening instrument to assess clinically relevant and everyday-life (e.g., economic, political, personal) anxieties. Furthermore, factors influencing these anxieties, correlations between clinical and everyday anxieties and, for the first time, anxiety during different stages of life were assessed in a representative sample of the general population (*N* = 2229). Around 30% of the respondents manifested at least one disorder-specific key symptom within 1 year (women > men), 8% reported severe anxiety symptoms. Two thirds of respondents reported minor everyday anxieties and 5% were strongly impaired, whereby persons with severe clinical symptoms were more frequently affected. A variety of potential influencing factors could be identified. These include, in addition to socioeconomic status, gender, general health, risk-taking, and leisure behavior, also some up to now little investigated possible protective factors, such as everyday-life mental activity. The observed effects are rather small, which, however, given the heterogeneity of the general population seems plausible. Although the correlative design of the study does not allow direct causal conclusions, it can, however, serve as a starting point for experimental intervention studies in the future. Together with time series from repeated representative surveys, we expect these data to provide a better understanding of the processes that underlie everyday-life and clinical anxieties.

## Introduction

Anxiety disorders are the most prevalent mental disorders. In Europe, their point prevalence is ~10%, 1-year prevalence 14%, and lifetime prevalence up to 29% (Michael et al., 2007; Wittchen et al., 2011; Baxter et al., 2013). Worldwide, anxiety disorders occur most frequently in high-income countries, but also in regions with current politico-military conflicts (Baxter et al., 2014). In both poor and rich countries, anxiety disorders are a major cause of disease-induced stress (“years of life lived with disability—YLDs”), ranking even higher than widely recognized widespread diseases, such as diabetes, chronic lung disease or arthrosis. Among mental disorders, anxiety disorders are surpassed only by depression, for which, however, they are key predictors (Baxter et al., 2014). Further epidemiological findings have shown pronounced subjective suffering, strongly limited work productivity and high comorbidity, especially together with other anxiety disorders and depression, but also with substance abuse (Michael et al., 2007). Prospective longitudinal studies show that anxiety disorders are a key risk factor for later occurring mental disorders (e.g., Beesdo et al., 2009; Trumpf et al., 2010; Kossowsky et al., 2013). Moreover, clinically significant anxieties can develop at a very early age (Seehagen et al., 2014) and often take a chronic course (Margraf and Poldrack, 2000; Seehagen et al., 2014). Anxiety disorders therefore play a substantial role in public healthcare policy. Despite their massive consequences and high chronicity, their treatment rate ranges only between 40 and 50% (Margraf and Poldrack, 2000; Wittchen and Jacobi, 2001; Lieb et al., 2003; Jacobi et al., 2004, 2014). Taken together, descriptive and analytical epidemiological studies on anxiety disorders are therefore of great interest. Among the identified influencing factors are gender as well the professional and socioeconomic status: Women, unemployed persons, housewives or househusbands, those with less education or in a poor financial situation are more frequently affected (for an overview cf. Lieb et al., 2003).

To assess epidemiological data, clinical interviews are the gold standard (such as, for example, CIDI, Kessler and Ustün, 2004 or DIPS, Schneider et al., 2012). However, clinical interviews are rather time consuming, rendering epidemiological studies complex and expensive. Although, anxiety disorders are, compared to other mental disorders, epidemiologically relatively broadly studied (Lieb et al., 2003), this may be one of the reasons why epidemiological studies are carried out considerably less often than experimental studies. Further, survey periods are often far apart, so that annual fluctuations in the frequency of occurrence cannot be examined. However, a more thorough view on the complex interplay of epidemiological factors would promote a better understanding of clinically significant anxiety and anxieties focusing on everyday-life issues (such as socioeconomic or political factors), as well as help to identify secular trends and possible correlations with macrosocial factors. For example, there are indications from North America that general anxieties have increased by approximately one standard deviation over the last half of the century (Twenge, 2000). In addition, correlations between habitual worrying (or brooding) and clinical anxiety have been found (Calmes and Roberts, 2007; McLaughlin et al., 2007). Generally speaking, episodes of subjectively experienced anxiety or fear (Zelenski and Larsen, 2000; Shapiro et al., 2001), and episodes of ruminative worries (Wells and Morrison, 1994; Szabó and Lovibond, 2002) are not uncommon in everyday life. To date, such general anxieties, fears and worries and their connection with clinical anxiety in the general population have been rarely investigated.

With the present study, we aimed to test a screening instrument which allows an efficient and comparatively inexpensive investigation of such questions in representative surveys among the general population. The screening instrument is attended to be used in settings where time and financial resources do not permit the use of full-blown clinical interviews. Our specific goal is to gain information about the frequency of clinical and everyday-life (non-clinical) anxieties as well as their risk factors, whereby the duration of the survey should not exceed 15 min. The instrument shall be used in an annual rhythm to investigate possible secular trends. Clinical anxieties are captured in two stages: First, the short questionnaire for anxiety disorders (KFA, Margraf, 1994), a short form of the German version of the Beck Anxiety Inventory (BAI, Margraf and Ehlers, 2007), will capture suffering from cross-disorder (disorder-unspecific) symptoms, which are predictive of an anxiety disorder. Thereby, the total frequency of people suffering from severe clinically relevant anxiety symptoms can be estimated. However, neither the short form nor the long form of the BAI distinguishes anxiety disorders according to DSM or ICD criteria (Margraf and Poldrack, 2000; Margraf and Ehlers, 2007). In a second stage, thus specific contents of clinically relevant anxieties are assessed by using the core questions of the Diagnostic Interview for Clinical Disorders (DIPS, Schneider et al., 2012). In addition to the information about the intensity and nature of clinical anxieties, the stress caused by non-clinical everyday-life anxieties is examined. Finally, demographic variables and other possible predictive factors for anxiety, as well as the intensity of anxieties during different stages of life are collected.

## Methods and materials

### Participants

The final sample of the present study consisted of *N* = 2229 German-speaking residents aged 18 or older within the Federal Republic of Germany. The sample is representative in terms of the distribution of the resident population in the federal states, household size, age groups as well as gender distribution. The survey was carried out in two subsamples with a mixed methods approach (recruited according to a common sampling system for telephone surveys, “Easy Sample,” Arbeitsgemeinschaft Deutscher Marktforschungsinstitute e.V., ADM, Germany). The sample response rates were 48% respectively 60%. A subsample of *n* = 1008 individuals was interviewed over the telephone, while a subsample of *n* = 1221 individuals completed the survey either online or using paper and pencil. Altogether, 1082 (48.6%) respondents were male and 1147 (51.4%) respondents female. The age of the respondents ranged between 18 and 98 years (*M* = 50.1, *SD* = 18.3). The distribution of the variables educational level, net household income and profession is shown in Table 1. Data collection took place between March 26th and May 3rd 2013 and was carried out by an institute for market and social research (USUMA GmbH, Berlin). The study was conducted in agreement with the Declaration of Helsinki and was approved by the ethics committee of the Faculty of Psychology at Ruhr-Universität Bochum.

**Table 1:** **Educational levels, occupations and net household income of the sample**.

**Sociodemographic feature**	**Category**	***n***	**% Total sample**
Education Level	Secondary modern/primary school without completed apprenticeship	87	3.9
	Secondary modern school/primary school with completed apprenticeship	311	14.0
	Secondary/middle/upper secondary/professional school/commercial school without A level	754	33.8
	A level/higher education entrance qualification	412	18.5
	University degree	619	27.8
	Still in school	34	1.5
Occupation	Simple jobs—unskilled/semi-skilled	137	6.2
	Skilled workers—journeyman/skilled worker qualification	259	11.6
	Employee without authority	474	21.2
	Employee with authority/executive employee	699	31.4
	Civil servant: low/middle-level service	53	2.4
	Civil servant: higher/upper-level service	173	7.8
	Self-employed person/ freelancer	228	10.2
	Self-employed farmer	17	0.7
	Without previous occupation	153	6.9
Net household income	Up to below € 500	20	0.9
	From 500 up to below 750 €	33	1.5
	From 750 up to below 1000 €	97	4.3
	From 1000 up to below 1500 €	263	11.8
	From 1500 up to below 2000 €	300	13.4
	From 2000 up to below 3000 €	529	23.7
	From 3000 up to below 4000 €	335	15.0
	4000 € and more	301	13.5

### Questionnaires

#### Short questionnaire for anxiety disorders (KFA)

For recording disorder unspecific (cross-disorder) anxiety symptoms, the Short Questionnaire for Anxiety Disorders (KFA, Margraf, 1994) was used. It represents a short form of the German version of the Beck Anxiety Inventory (BAI, Margraf and Ehlers, 2007), which was successfully used in previous studies to estimate prevalence rates (Margraf and Poldrack, 2000). Like the long form, the short questionnaire of six items assesses the existence of a number of mainly physical anxiety symptoms within the last 7 days. Joint occurrence of these symptoms indicate the likelihood of an anxiety disorder (wobbliness in knees or legs, dizzy or lightheaded, shaky or unsteady, hands trembling, scared, feeling of faintness)^1^. Participants indicate on a 4-point scale how much they suffered from the respective symptom (0 = not at all, 1 = mildly, 2 = moderately, it was very uncomfortable but I could bear up against it, 3 = severely, I could hardly bear up against it). The sum score of the scale can vary between 0 and 18 points, where values between 0 and 3 are interpreted as “no disorder,” values between 4 and 6 as “potential disorder” and values greater than 6 as “definite disorder.” A principal component analysis with varimax rotation confirmed the single-factor solution, internal consistency of the scale in the present study was *CR*α = 0.83.

#### Specific symptoms of anxiety disorders

The occurrence of specific anxiety symptoms according to DSM-IV-TR was assessed with seven questions closely following the basic questions of DIPS (Schneider and Margraf, 2011) concerning the core symptoms of various anxiety disorders. Assessing core symptoms is a time-efficient screening method with good diagnostic accuracy (Wittchen et al., 1999). In the present study, the respondents were asked whether the relevant symptoms occurred within the last 12 months, whether the symptoms were present longer than 12 months, or whether they had never occurred.

#### Anxiety during different stages of life

The distribution of anxiety during different stages of life was assessed by asking the respondents to specify the intensity of anxiety they experienced during different stages of life (scale 0–3, see section on KFA). The stages of life were chosen according to Havighurst (1948/1972): early childhood (2–4 years), early school age (5–7 years), middle school age (8–12 years), adolescence (13–17 years) and late adolescence (18–22 years), as well as early (23–30 years), middle (31–50 years) and late adulthood (from 51 years on).

#### Anxieties regarding different aspects of everyday-life

Regarding the intensity of anxiety related to aspects of everyday-life, participants responded on a scale from 0 to 3, analogously to KFA, if they currently feel anxious or worry about the following aspects of life: (1) school, studies, work, (2) family, (3) friends, (4) neighbors/neighborhood, (5) finances, (6) health, (7) Internet, social networks, (8) personal future or the future of their children, (9) war, terrorism, (10) environmental disasters, (11) general economic situation in Germany and Europe, (12) general political situation in Germany and Europe. In order to reduce the dimensionality of the scale, a principal component analysis with varimax rotation was carried out with the answers to these 12 items; the number of factors was determined by means of the scree plot. This resulted in a three-factor solution with the factors “political and economic environment” (Items 9, 10, 11, 12, *CR*_Scale_ = 0.86), “own person and family” (Items 1, 2, 5, 6, 8, *CR*_Scale_ = 0.72) and “extended personal environment” (Items 3, 4, 7, *CR*_Scale_ = 0.47, without Item 7 *CR*_Scale_ = 0.53). Mean scale values were generated for further analysis of the individual scales (range: 0–3 each).

#### Leisure activities and self-view

To assess the frequency of leisure activities and media consumption, respondents were asked to specify on a 5-point scale (0 = never, 1 = daily, 2 = weekly, 3 = monthly, 4 = yearly) how often they carry out the following activities (1) meeting friends and acquaintances (2) pursuing intellectual activities, such as reading newspapers or books, going to the theater or playing music, (3) watching TV, videos or DVDs, (4) doing sports, (5) playing games on the computer or games console, (6) using the Internet^2^.

To assess self-view, participants were asked to estimate on a scale from 0 to100 how they experience their present state of health, their willingness to take risks and their attractiveness compared to other persons.

#### Demographic variables and socioeconomic status

Apart from age, sex and household size (number of persons permanently living in the household), current occupation (11-level categorical selection), highest educational level (6-level categorical selection), and net household income were recorded in order to determine socioeconomic status. Additionally, respondents were asked to specify if they are currently employed (7-level categorical selection) and their present marital status. All categories used are included in Table 1. Categories of a low, middle, and high socioeconomic status (SES) were generated by means of the variables occupation, net household income and level of education, (for a detailed description see Lampert et al., 2013a,b). The current survey considered only the persons who gave answers to all three variables (*n* = 1736, corresponding to 78% of the total sample). The three middle quintiles were summarized into the category “middle socioeconomic status,” while the lowest quintile created the category “low” and the highest quintile “high socioeconomic status” (according to Lampert et al., 2013a,b).

### Data reduction and data analysis

In order to assess participants' general symptom load, descriptive statistics for anxieties/worries concerning aspects of everyday-life, clinical anxieties and anxiety intensity during different stages of life were calculated and the frequencies for the occurrence of specific clinical anxieties were assessed. Then, all respondents who achieved a KFA sum score of ≥7, were categorized as severely suffering from clinically relevant but disorder unspecific anxiety symptoms. To additionally determine disorder specific symptom load, for these persons, the occurrence of disorder specific anxiety symptoms within the last year was assessed (i.e., panic attacks, agoraphobic fear, etc.).

*T*-tests, or if necessary due to violations of distribution, Mann-Whitney-U tests or Wilcoxon-tests, were used to examine gender influence on the occurrence of disorder-specific and unspecific clinically relevant symptoms, the intensity of the anxieties during different stages of life as well as anxieties concerning aspects of everyday-life. In addition, Spearman rank correlations between different anxieties and the variables occupation, education, net household income and leisure behavior, as well as Pearson correlations between age and self-view of respondents, on the one hand, and general anxieties and intensity of cross-anxiety symptoms, on the other hand, were calculated.

For quantifying the influence of age, occupation, education and net household income on the occurrence of severe clinical symptoms, odds ratios were calculated. To estimate non-linear correlations, additional χ^2^ tests were used in order to examine if the frequency of severe anxiety symptoms differs between the categories high, middle and low socioeconomic status. For the same purpose, ANOVAs with planned contrasts were conducted to compare the average anxiety intensity on the three scales of anxieties/worries concerning everyday-life between the three categories of socioeconomic status. In order to examine the effect of employment status, odds ratios were calculated under the control of the influencing factors of age and gender by means of logistic regression. Finally, the relation between anxieties/worries concerning everyday-life and cross-disorder anxiety symptoms was examined by means of point-biserial correlations and the intensity of anxieties/worries concerning everyday-life between persons with and without an anxiety disorder was compared by using *t*-tests or, if necessary due to violations of distribution, with Mann-Whitney-U tests. A significance level of α = 0.05 was established for all statistical tests. In the case of variance-analytical methods, the effect size f was calculated; for *t*-tests we calculated the effect size d. All correlations were corrected according to Bonferroni.

## Results

### Clinically significant anxieties

Altogether, the current load of cross-disorder clinical anxiety symptoms was low, whereby women showed a higher load than men [KFA sum score of the total sample *M* = 1.88, *SD* = 3.01; men: *M* = 1.61, *SD* = 2.85; women: *M* = 2.14, *SD* = 3.13; *t*_(2227)_ = 4.19, *p* < 0.001, *d* = 0.18]. As Table 2 shows, the specific core symptoms occurred in up to one-third of all respondents within the last year. Anxiety of social and achievement situations (33.7% of the total sample), panic attacks and phobic anxieties (24.1 resp. 24.0%) were most commonly reported. About one-fifth of all respondents suffered in the course of the last year from obsessive thoughts/ compulsive behavior (19.6%), or uncontrollable worries (19.0%). If one extends the period considered to the entire lifetime, then the values are approximately twice as high. Women are, with the exception of social anxiety, traumatic experiences and obsessive thoughts/ compulsive behavior more frequently affected than men (Table 2).

**Table 2:** **Occurrence of the core symptoms of clinically significant anxieties**.

	**Occurrence of core symptoms over the last 12 month (%)**					**Occurrence of core symptoms over the lifespan (%)**				
	**Total sample**	**Men**	**Women**	**With severe anxiety symptoms**	**Without severe anxiety symptoms**	**Total sample**	**Men**	**Women**	**With severe anxiety symptoms**	**Without severe anxiety symptoms**
Panik	24.1	19.2^*^	28.7	61.6	21.2	44.7	40.9^*^	48.2	72.7^*^	42.9
Agoraphobic fear	11.1	8.4^*^	13.7	31.2	9.7	21.6	16.3^*^	26.6	40.5^*^	20.4
Phobic fear	24.0	18.5^*^	29.3	39.4	23.3	44.8	37.6^*^	51.6	57.7^*^	44.7
Social anxiety	33.7	34.5^n.s.^	33.0	43.1	33.5	64.1	64.2^n.s.^	64.1	68.4^n.s.^	64.9
Trauma	5.2	4.6^n.s.^	5.8	10.9	5.1	24.4	22.9^n.s.^	25.9	42.9^*^	23.5
Compulsive behavior or thoughts	19.6	19.3^n.s.^	20.0	38.1	18.4	30.9	30.3^n.s.^	31.4	52.3^*^	29.7
Generalized anxiety	19.0	14.2^*^	23.4	50.6	16.7	37.6	30.3^*^	44.3	64.2^*^	36.1

*Shown are the percentage frequencies in the total sample, in men and women as well as in persons with resp. without severe anxiety symptoms (KFA sum score ≥ 7)*.

Comparison men vs. women or persons with vs. without severe symptoms

* p < 0.05;

n.s. *p > 0.05*.

A total of 179 respondents (8% of the total sample) achieved a KFA sum score of ≥7. Hence, they fulfilled the previously defined criterion for severely suffering from anxiety symptoms. Women were more frequently affected than men (men: *n* = 72; 6.7%; women: *n* = 107; 9.3%; χ^2^_*df*__1_ = 5.39, *p* = 0.02, *OR* = 1.43, *p* = 0.023, *KI* = 1.05–1.96). During the last 12 months, persons suffering severely from anxiety symptoms most frequently experienced core symptoms of panic disorder and generalized anxiety disorder (62 or 51% of the persons with severe anxiety symptoms), followed by core symptoms of social anxiety disorder (43%), specific phobia (39%) and obsessive-compulsive disorder (38%). The core symptoms of agoraphobia (31%) and trauma (11%) occurred slightly less frequently. Accordingly higher values apply for the occurrence of symptoms during periods longer than 12 months (lifetime). In total, 61% of the persons with severe anxiety symptoms reported the occurrence of two or more core symptoms of specific anxiety disorders within the past year and only 39% the occurrence of only one core symptom. Moreover, all disorder-specific core symptoms occur, as expected, significantly more frequently within the group of persons with severe anxiety symptoms than in persons without severe anxiety symptoms (Table 2).

Related to the entire sample (*N* = 2229), the combination of severe anxiety symptoms and panic symptoms or symptoms of generalized anxiety disorder occurred most frequently (4.9 and 4.1% of the total sample), followed by the combination of severe anxiety symptoms and symptoms of social anxiety disorder (3.4%), specific phobia (3.2%), and obsessive-compulsive disorder (3.1%). Symptoms of agoraphobia (2.5%) and post-traumatic stress disorder (0.9%) occurred less frequently among persons with severe anxiety symptoms.

### Influencing factors on clinically significant anxieties

#### Disorder-specific core symptoms and cross-disorder symptoms

Table 3 gives an overview of the correlations between cross-disorder anxiety symptoms, core symptoms and possible influencing factors. Among sociodemographic variables, apart from gender, particularly socioeconomic status (especially income) is interrelated with clinically significant anxieties. Income is significantly negatively correlated with cross-disorder anxiety symptoms (KFA sum score). Similar correlations can be also found for currently practiced profession and education. Regarding disorder-specific anxiety symptoms, a significantly negative correlation between age and core symptoms of social anxiety is notable.

**Table 3:** **Correlation coefficients and odds ratios (OR) for the correlation between sociodemographic variables, leisure activities and self-view with clinically significant anxieties**.

	**Panic symptoms**	**Agoraphobic symptoms**	**Phobic symptoms**	**Symptoms of social phobia**	**Trauma**	**Compulsive symptoms**	**Symptoms of generalized anxiety**	**KFA- sum score**	**Odds ratios for severe anxiety symptoms**	
									***OR***	***KI***
Occupation	−0.048	−0.019	−0.007	−0.102^*^	0.012	−0.023	−0.042	−0.087^*^	0.83^*^	0.73–0.95
Income	−0.099^*^	−0.058	−0.054	0.093^*^	−0.052	−0.114^*^	−0.081^*^	−0.196^*^	0.74^*^	0.66–0.83
Education	−0.028	−0.014	−0.043	0.108^**^	−0.044	−0.065^*^	−0.061^*^	−0.100^*^	0.83^*^	0.74–0.93
Age	0.008	−0.010	−0.065^*^	−0.391^*^	−0.002	0.032	−0.026	0.055	1.01	1.00–1.02
Meeting frieds	−0.040	−0.042	−0.027	0.088^*^	−0.014	−0.065^*^	−0.047	−0.074^*^	0.76^*^	0.63–0.91
Intellectual activities	0.005	−0.013	−0.039	−0.120^*^	0.004	−0.036	−0.046	−0.109^*^	0.76^*^	0.66–0.88
Watching TV	0.012	−0.020	0.022	−0.088^*^	0.001	−0.021	0.005	−0.016	0.91	0.74–1.12
Doing sports	−0.012	−0.031	−0.031	0.062^*^	−0.025	−0.050	−0.034	−0.105^*^	0.90	0.81–1.01
Computer gaming	0.045	−0.006	−0.012	0.144^*^	−0.009	−0.009	−0.014	0.007	1.14^*^	1.03–1.26
Using the internet	−0.005	−0.031	0.026	0.237^*^	0.009	−0.099^*^	−0.010	−0.102^*^	0.78^*^	0.69–0.87
Self-rated health	−0.238^*^	−0.150^*^	−0.163^*^	0.032	−0.076^*^	−0.134^*^	−0.168^*^	−0.411^*^	0.96^*^	0.95−0.96
Self-rated risk-taking	−0.055	−0.087^*^	−0.090^*^	−0.021	−0.022	−0.096^*^	−0.094^*^	−0.087^*^	0.99	0.90–1.00
Self-rated attractiveness	−0.059	−0.070^*^	−0.092^*^	−0.003	−0.052	−0.081^*^	−0.104^*^	−0.128^*^	0.98^*^	0.97–0.99

* p < 0.05;

** *p < 0.01; p-values are Bonferroni corrected*.

Also leisure activities show clear correlations with cross-disorder anxiety symptoms. For the aspects of meeting friends, intellectual activities, doing sports and using the Internet, the correlation is significantly negative. By contrast, there are only weak correlations with disorder-specific core symptoms. Anxieties concerning social and evaluation situations are an exception featuring a robustly positive correlation with Internet use and computer gaming as well as a negative correlation with intellectual activity.

Further significantly negative correlations with cross-disorder symptoms and most of the core symptoms of specific anxiety disorders are shown for self-evaluation of physical health, risk-taking and attractiveness.

#### Severe anxiety symptoms

Analogous with the influence of the socioeconomic status on the occurrence of anxiety symptoms, socioeconomic status correlated with severe suffering from anxiety symptoms (defined by the KFA sum score ≥7). After all, 15.7% of the respondents with a low socioeconomic status are affected severely. This value is significantly higher than in persons with a middle (6.5%, χ^2^_*df*__1_ = 20.40, *p* < 0.001) or high socioeconomic status (4.3%, χ^2^_*df*__1_ = 17.58, *p* < 0.001) where the frequency of the occurrence of anxiety disorders was equally high (χ^2^_*df*__1_ = 1.65, *p* > 0.10). Also the logistic regression indicates a strong correlation between a low socioeconomic status and the occurrence of severe anxiety symptoms (*OR* = 2.37, *p* < 0.001, *KI* = 1.47–3.83).

The logistic regression with the three variables constituting socioeconomic status (education, occupation, income) primarily shows a significantly negative correlation between income and the occurrence of severe anxiety symptoms. The negative correlations with education and type of employment are, in contrast, less pronounced (see Table 3). Generally, the probability of severe anxiety symptoms decreases with full-time (*OR* = 0.49, *p* < 0.001, *KI* = 0.34–0.72) or part-time employment (*OR* = 0.46, *p* = 0.004, *KI* = 0.27–0.78). On the other hand, increased probabilities can be found in retired persons (*OR* = 1.71, *p* = 0.045, *KI* = 1.01–2.88) as well as in students and school pupils (*OR* = 3.58, *p* < 0.001, *KI* = 2.13–6.02). In contrast, professional training (*OR* = 0.92, *p* = 0.803, *KI* = 0.31–2.78), unemployment (*OR* = 1.67, *p* = 0.122, *KI* = 0.87–3.18) or being a housewife/househusband (*OR* = 1.54, *p* = 0.071, *KI* = 0.96–2.46) seem to have no direct influence on the probability of an anxiety disorder.

Negative correlations are found between self-evaluated health as well as self-evaluated attractiveness and occurrence of severe anxiety symptoms. Correlations between leisure behavior and the occurrence of severe anxiety symptoms are evident, too. Thus, the leisure activities “meeting friends,” “intellectual activity” as well as “Internet use” correlate negatively with the occurrence of severe anxiety symptoms, whereas “computer gaming” correlates positively (see Table 3).

### Anxieties regarding different aspects of everyday-life

Table 4 shows the average level of anxiety of female and male participants with regard to the different aspects of life. Again, the level of stress in the general population is overall rather low, whereby the aspect “extended social environment” has the lowest anxiety scores. Overall, about two thirds of the respondents show no or little anxiety concerning the political and economic environment (65.2%, *M* = 0.47, *SD* = 0.39) and their own person and family (64.3%, *M* = 0.53, *SD* = 0.33). Another third of the respondents expressed little to moderate anxieties in both areas (political and economic environment: 27.6%, *M* = 1.60, *SD* = 0.27; own person and family: 30.8%, *M* = 1.49, *SD* = 0.27). For anxiety regarding the extended personal environment, 94.8% reported no or little anxiety (*M* = 0.21, *SD* = 0.31); only 4.4% reported little to moderate anxiety (*M* = 1.53, *SD* = 0.25). However, 6.9% (moderate anxiety *M* = 2.56, *SD* = 0.29) of the respondents report moderate to strong anxiety for political and economic environment and 4.7% (moderate anxiety *M* = 2.39, *SD* = 0.22) for their own person and family. The rate for moderate to strong anxiety for extended personal environment account only for 0.3% (moderate anxiety *M* = 2.51, *SD* = 0.18).

**Table 4:** **Means and standard deviations of everyday anxieties (of individual aspects of life and scales)**.

**Scale**	**Item**	**Item**						**Scale**						
		**Total sample**		**Men**		**Women**		**Total sample**			**With severe anxiety symptoms**		**Without severe anxiety symptoms**	
		***M***	***SD***	***M***	***SD***	***M***	***SD***	***M***	***SD***	**CRα (Skala)**	***M***	***SD***	***M***	***SD***
Political and economic environment	War/Terrorism	0.80	0.90	0.63	0.83	0.96	0.9^*^	0.93	0.76	0.9	1.29	0.88	0.90	0.74
	Environmental disasters	0.79	0.88	0.62	0.80	0.95	0.92^*^							
	Economy	1.11	0.91	1.07	0.93	1.15	0.90^n.s.^							
	Politics	1.00	0.91	0.96	0.92	1.04	0.90^n.s.^							
Own person and family	Occupation	0.74	0.88	0.81	0.89	0.68	0.88^*^	0.92	0.63	0.7	1.37	0.75	0.88	0.60
	Family	0.81	0.90	0.70	0.86	0.90	0.93^*^							
	Finances	0.90	0.93	0.88	0.92	0.92	0.94^n.s.^							
	Health	1.10	0.93	1.03	0.92	1.15	0.93^*^							
	Future	1.03	0.92	0.92	0.89	1.14	0.94^*^							
Extended social environment	Internet/social networks	0.21	0.57	0.21	0.57	0.21	0.57^n.s.^	0.27	0.43	0.5	0.50	0.57	0.26	0.41
	Friends	0.36	0.64	0.30	0.59	0.42	0.68^*^							
	Neighbors	0.23	0.56	0.22	0.55	0.25	0.57^n.s.^							

* Comparison women vs. men p < 0.05;

n.s., *Comparison women vs. men p > 0.05*.

**Figure 1:**
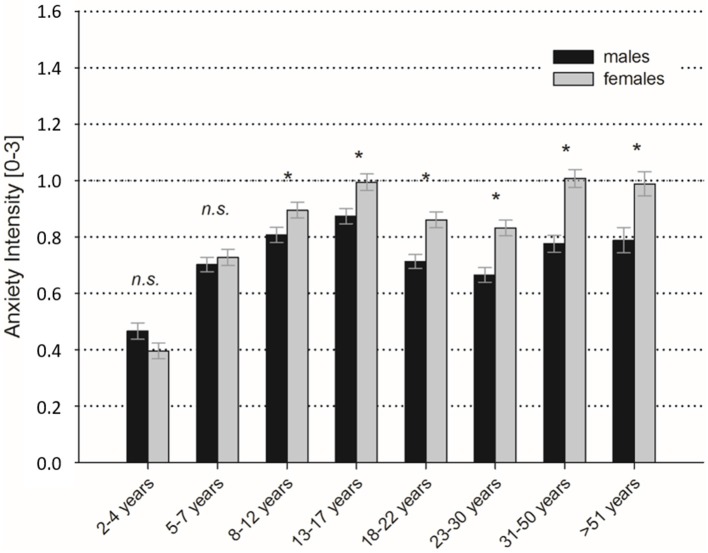
**Mean values (±SEM) of anxiety intensity during different stages of life separated by gender**. (^*^p < 0.05).

With regard to the general suffering from anxiety in different stages of life, we found overall low to moderate levels of stress in the total sample (see Figure 1). However, gender differences become apparent in general anxieties as well. In all three subscales, women show higher values than men. Furthermore, the higher suffering from anxiety of females (see Section Clinically Significant Anxieties) begins already in middle school age. While the anxiety intensity in early childhood and in early school age does not significantly differ (both *p* > 0.10), there are significant differences in all later life stages between men and women (all *p*'s < 0.05). In addition, suffering from anxiety varies significantly between the stages of life. Suffering increases from early childhood to adolescence (early childhood vs. early school age, *z* = 10.7, *p* < 0.001, *d* = 0.27; early school age vs. middle school age, *z* = 7.2, *p* < 0.001, *d* = 0.15; middle school age vs. adolescence, *z* = 4.6, *p* < 0.001, *d* = 0.09), then decreases (adolescence vs. late adolescence, *z* = −8.6, *p* < 0.001, *d* = 0.15) and remains constant on this level over late adolescence and early adulthood (late adolescence vs. early adulthood, *z* = −0.6, n.s.). Thereafter, suffering increases again into middle adulthood and then remains constantly high to late adulthood (early adulthood vs. middle adulthood, *z* = 7.2, *p* < 0.001, *d* = 0.18; middle adulthood vs. late adulthood, *z* = 1.8, n.s.).

### Influencing factors for the occurrence of general anxieties

Besides gender, socioeconomic status and the age of the respondents influences the manifestation of anxieties in different aspects of life (see Table 5). Whereas age is significantly negatively associated with anxieties in the areas of person/ family and extended social environment, a significantly positive correlation is evident for anxieties concerning the political and economic environment. Education, income and current occupation were consistently negatively correlated with general anxieties. Women are more strongly affected by anxieties than men in all three aspects of life (political and social environment: *z* = 6.1, *p* < 0.001, *d* = 0.27; own person and family: *z* = 4.2, *p* < 0.001, *d* = 0.15; extended social environment: *z* = 3.5, *p* = 0.001, *d* = 0.12).

**Table 5:** **Correlation coefficients for the correlation between the three scales of everyday anxieties as well as sociodemographic variables, leisure activities and self-view**.

	**Political and economic environment**	**Own person and family**	**Extended social environment**
Occupation	0.007	−0.082^*^	−0.027
Income	−0.147^*^	−0.107^*^	−0.034
Education	−0.129^*^	−0.045^*^	−0.017
Age	0.159^*^	−0.103^*^	−0.089^*^
Meeting friends	−0.096^*^	−0.080^*^	0.019
Intellectual activities	0.010	−0.128^*^	−0.041
Watching TV	0.048^*^	−0.004	0.011
Doing spots	−0.003	−0.014	−0.005
Computer gaming	−0.037	0.060^*^	0.074^*^
Using the internet	−0.121^*^	0.052^*^	0.059^*^
Self-rated health	−0.145^*^	−0.268^*^	−0.114^*^
Self-rated risk-taking	−0.095^*^	−0.038	−0.012
Self-rated attractiveness	−0.088^*^	−0.092^*^	−0.049

* *p < 0.05, p-values are bonferroni corrected*.

ANOVAs comparing the intensity of general anxieties between the levels of socioeconomic status confirm the linear correlations for anxieties concerning the political and economic environment [*F*_(2, 1771)_ = 17.67, *p* < 0.001, *f* = 0.14]. Persons with a low socioeconomic status show significantly stronger anxieties than persons with a middle socioeconomic status (*p* < 0.05). Persons with a high status show the lowest anxiety intensity (comparison with middle status and low status *p* < 0.05). In case of anxieties concerning one's own person and family [*F*_(2, 1771)_ = 17.63, *p* < 0.001, *f* = 0.14] and the extended personal environment [*F*_(2, 1771)_ = 6.29, *p* = 0.002, *f* = 0.08], however, persons with a middle and high status have similar anxiety levels. (*p* > 0.05). Persons with a low socioeconomic status experience higher anxiety intensity compared to those with a high or middle status (both *p* < 0.05).

Furthermore, self-evaluations of health and attractiveness correlate significantly negatively with all three subscales of general anxieties. Regarding leisure behavior, we found significantly positive correlations between computer gaming and anxieties concerning family and social environment, while intellectual activity is negatively associated with anxieties regarding the family. Moreover, the use of the Internet correlates negatively with anxieties about the political and economic environment.

### Correlation between clinical and everyday-life anxieties

Assuming that there is a continuum of anxiety, clinical and general anxieties are expected to correlate with each other. Our data in fact show significant correlations of cross-disorder anxiety symptoms with all three areas of anxieties/worries concerning aspects of everyday-life (political and economic environment *r* = 0.154, *p* < 0.001, own person and family *r* = 0.297, *p* < 0.001, extended personal environment, *r* = 0.173, *p* < 0.001). The fact that persons with severe anxiety symptoms (KFA sum score ≥7) show a significantly higher levels of stress in all three areas of everyday-life (political and economic environment: *z* = 5.9, *p* < 0.001, *d* = 0.49; family and own person: *z* = 8.4, *p* < 0.001, *d* = 0.72; extended personal environment: *z* = 6.5, *p* < 0.001, *d* = 0.49) points in the same direction (see also Table 4).

## Discussion

The first objective of this study was to test an efficient and inexpensive survey instrument for clinically significant and everyday anxieties in the general population. Furthermore, we wanted to examine influencing factors on anxieties, correlations between clinical and anxieties/worries concerning aspects of everyday-life as well as the development of anxiety during different stages of life.

### Valuation of the screening instrument and assessment of frequency of anxieties

Our results correspond well with the findings of previous epidemiological studies on prevalences and influencing factors of anxiety disorders. Moreover, the survey fit the scheduled time frame (~15 min per person). The psychometric properties of the used short questionnaire as well as two of the three scales for the everyday-life anxieties are good. Thus, the questionnaire reliably assesses anxieties concerning political and economic environment (CRα = 0.9), as well as one's own person and family (CRα = 0.7). The scale “extended personal environment” turned out to be less reliable (CRα = 0.5). One possible reason could be that the item assessing Internet use/social networks did not reliably assess the diverse aspects of Internet use in the general population (cf. Beutel et al., 2011). As a consequence, the items assessing Internet use require substantial revision for future versions of the questionnaire. At the moment, the informative value of results of the extended personal environment subscale might be limited.

However, taken as a whole, the first objective of the study was fulfilled; the instrument has been proven reliable in practical trials. Further studies have to clarify the conformity of our self-evaluation report inventory with diagnoses based on structured clinical interviews. According to previous studies, however, a high level of conformity might not necessarily be expected (e.g., Wittchen et al., 1999).

In total, within a period of 1 year approximately one third of the general population is affected by at least one core symptom that is specific for an anxiety disorder. Especially social anxieties (around 34%) as well as panic attacks and phobic anxieties (24% each) occur frequently. About one fifth of the general population reports obsessive thoughts or compulsive acts (20%) and uncontrollable worries (19%). Agoraphobic anxieties (11%) and traumatic experiences (5%) occurred slightly less frequently. If one extends the period considered to the entire life span, the frequency of one or more specific core symptoms increases to two thirds of the general population. However, by far not all persons showing one or more core symptoms specific for anxiety disorders over a very limited period of time will develop a full disorder. On the one hand, the general population shows only a low stress level concerning cross-disorder anxiety symptoms, proven by the comparatively weak KFA sum scores. On the other hand, the current prevalence of severe anxiety symptoms is, according to our data, at only 8%, and thus significantly lower than the frequency of specific core symptoms. These data thus confirm epidemiological studies showing that subclinically significant specific symptoms of anxiety disorders appear frequently (Margraf and Ehlers, 1988; Carter et al., 2001; Kessler et al., 2006; Beesdo et al., 2008), but often do not necessarily develop into clinically relevant disorders.

On the other hand, our data show that currently 8% of the general population are very highly stressed by clinically significant symptoms which are very likely to fulfill the entire set of criteria for an anxiety disorder. Our estimation of the prevalence of severe anxiety symptoms meets our expectations based on previous studies conducted with the KFA (cf. Margraf, 1994) and are comparable to point and 4-week prevalences of anxiety disorders in representative epidemiological studies (e.g., Jacobi et al., 2004). Taken together evidence from the literature suggests that the current method reliably estimates the prevalence of severe anxiety symptoms.

With regard to specific anxiety symptoms, our estimates are slightly above the prevalence rates reported in previous studies (for an overview see Lieb et al., 2003). Particularly the scores for symptoms of panic disorder are strikingly high (estimate in the present study: 4.9%). Although it has been previously shown (Wittchen et al., 1999) that basic questions about the core symptoms of a disorder have a generally satisfactory diagnostic accuracy, it can be assumed that basic questions about panic disorder show insufficient specificity (cf. Margraf and Ehlers, 1988). It seems plausible that the high frequency values for panic symptoms can be explained by the high comorbidity between the occurrence of panic attacks and other anxiety symptoms. For example, around 45% of persons who have experienced a panic attack, fulfill the criteria for the diagnosis of another anxiety disorder (Kessler et al., 2006). The inquiry of additional diagnostic criteria could be beneficial in this case.

The level of stress of the general population due to anxieties/worries concerning aspects of everyday-life is generally low. This applies particularly to the extended social environment. Activity on the Internet/social networks, meeting friends and neighbors belong to this aspect of life. Here, ~95% of the respondents show no or little anxieties and only around 1% report moderate to strong anxiety. In addition, two thirds of the respondents report little to no anxieties concerning the economic and political environment as well as their own person and family. Moderate to strong anxieties appear, on the other hand, in about 5% (personal environment and own person/family) and 7% (economic and political environment) of the respondents.

### Influencing factors on clinical and everyday anxieties

#### Sociodemographic variables

Correlations with sociodemographic variables are among the potential influencing factors. Generally, women reported specific clinical core symptoms more frequently (exception: social anxieties, traumatic experiences, and obsessive thoughts/compulsive acts) and fulfilled more frequently the criterion for severe anxiety symptoms than men. Moreover, women show stronger anxiety than men in all three aspects of everyday-life. These differences seem to develop with adolescence and to continue throughout the entire adulthood. In early childhood and early school age (until ~8 years), we found, in contrast, no difference between boys and girls. Taken together, these data thus support previous reports showing anxiety related gender differences in school aged children (Ollendick, 1983) and epidemiological studies showing consistently that women are more frequently affected by most anxiety disorders (i.e., Fredrikson et al., 1996; McLean and Anderson, 2009; McLean et al., 2011) and report greater burden of illness (McLean et al., 2011). However, due to our cross-sectional design, the present findings requires further verification.

Our data further extend previous findings by showing that gender differences cover a broad range of anxiety domains including clinically relevant as well as everyday life anxieties. Our data provide first reference points, although the cross-sectional design limits the significance of our results. In sum, the development of everyday-life anxieties over the lifespan is yet not sufficiently investigated and further research is needed to clarify etiological factors. Initial evidence, however, supports the notion that gender differences may emerge from a complex interplay between a diverse range of vulnerability factors including genetic risks, environmental influences and personality traits (review in McLean and Anderson, 2009).

Low socioeconomic status was found to be a risk factor. In persons with severe anxiety symptoms, the ratio of low socioeconomic status is significantly higher than middle or high status. Income in particular seems to be the best predictor for the occurrence of severe anxiety symptoms. Furthermore, regardless of the kind of the occupation—current employment situation has an influence: Persons with part-time or full-time employment demonstrate a lower probability for severe anxiety symptoms, whereas retired/pensioned respondents, on the other hand, show a higher probability. These results confirm previous studies (overview in Lieb et al., 2003), in which correlations with sociodemographic factors were found. The pronounced correlation between present school-/or university education and the occurrence of severe anxiety symptoms is striking: University students and school pupils seem to be exposed to a significant risk. For example, two thirds of university students complain about doubts regarding their studies, disorientation and impairment of the well-being due to hectic and stress (Stock and Krämer, 2000). In addition, a sharp increase in the frequency of nervousness because of exams was reported recently (Holm-Hadulla et al., 2009).

As in case of clinical anxieties, persons with a low socioeconomic status have significantly stronger worries concerning everyday-life than persons with a high or middle status. This extends previous research (c.f., for example Lieb et al., 2003) and shows that besides being strained by clinically relevant anxieties, people with low socioeconomic status worry more about multiple facets of life.

#### Self-evaluation and leisure activities

Self-evaluations of health, risk-taking and appearance are associated with the occurrence of clinical anxieties. This effect is most pronounced for subjective general health status, which correlates negatively with almost all clinical anxieties (exception: social anxieties), with strongest associations to panic attacks (*r* = −0. 24) and the sum score of the KFA (*R* = −0. 41).

Significantly lower, although still clear, are the negative correlations of clinically relevant anxieties with subjective risk-taking and attractiveness (*r* = −0.07 and *r* = −0.13). Remarkably, the correlations with the frequency of panic attacks, symptoms of social phobia and traumas are not significant. In general, comorbid anxiety disorders appear in persons with chronic somatic disorders twice as often as in persons without somatic stress (Klesse et al., 2008). However, the existence of specific somatic diseases was not investigated in our study, and, as we have pointed out earlier, our correlative design does not allow causal conclusions.

In contrast to personal self-evaluations and sociodemographic variables, leisure activities show only few significant correlations with clinical anxieties. Exception again are symptoms of social phobia which correlate negatively with intellectual activity and watching TV, but positively with Internet use and playing on the computer (*r* = 0.24 and *r* = 0.14), and weakly positive with meeting friends and doing sports (*r* = 0.09 and 0.06). The sum score of the KFA as a cross-disorder anxiety indicator correlates, in contrast, negatively with most of the estimates concerning leisure behavior. The negative correlations with meeting friends, intellectual activity and doing sports (*r* between −0.07 and −0.11) are in line with previous studies which have discussed these activities as potentially protective factors for the occurrence of anxiety disorders (see e.g., Egle et al., 1997; Steinhausen and Metzke, 2001). Interestingly, also the use of the Internet correlates negatively with the KFA sum score (*r* = −0.10), which contradicts the tenor of the public debate and among the educated middle-class as well as previous research showing that extended use of the Internet might constitute a risk factor for mental disorders (Yau et al., 2013). This finding clearly suggests that Internet use *per se* might not constitute a risk factor for the occurrence of mental health problems. Rather, the current data indicate that Internet use might also be a protective factor, possibly due to the fact that mental-health promoting activities such as intellectual or social activities can also be carried out using the Internet. A more differential picture is needed to clarify the specific relationship between problematic Internet use and mental health.

Concerning everyday-life anxieties, strongest correlations were found for self-evaluations, while leisure activities show more heterogeneous correlations. Most pronounced are correlations with anxieties concerning one's own person and family or the political and economic environment (10 or 9 of 13 correlations significant). In case of anxieties concerning the extended personal environment, in contrast, only 4 of 13 correlations reach the significance level corrected according to Bonferroni. However, the correlations with the three thematic aspects of everyday anxieties do not in all cases point in the same direction. For example, anxieties about the political and economic environment increase with age (*r* = 0.16), but anxieties about one's own person/family decrease (*r* = −0.10). With increasing Internet use, politicoeconomic anxieties decrease (*r* = −0.12), but anxieties in the two other thematic areas increase slightly (*r* = 0.05 and 0.06). The contrast between Internet use and watching TV is also striking: While the former is accompanied by less politic/economic anxiety, the correlation for the latter is weak, but significantly positive. Intellectual activity showed a special effect in its association with less personal/familiar anxiety, while there were no correlations regarding doing sports. Further, playing on the computer correlates with slightly higher anxiety scores in this dimension. The partially contrary correlations in the context of computer gaming, Internet and watching TV show that a general category “media use” is not feasible.

#### General considerations on influencing factors

Since we corrected all significances according to Bonferroni and the sample size of *N* > 2000 guarantees sufficient statistic power, smaller effects described here can also be taken seriously. It seems plausible to us that in the case of potential influencing factors on clinical anxieties, a variety of smaller effects become evident. It is likely that larger effects would have already been identified in previous studies. Of course, the restriction of the correlative design applies here, too. Nonetheless, the present data provide reference points for the planning of experimental follow-up studies, where the systematic examination of interventions to reduce risk factors or to increase protective factors may substantiate causal statements.

Finally, a methodological limitation might arise from the assessment of leisure activities. Although the questions assessing leisure activities were carefully chosen according to previously published work, some of the categories might not be entirely selective. Today, for example, newspapers and books may be read on the Internet, theater performances watched on television, videos or DVDs, and music played on the computer or game consoles. Thus, in future work, these categories should be revised to more closely cover the current use of new medias.

### Relations between clinical and everyday-life anxieties

Clinical and everyday-life anxieties show a significant overlap: The persons that fulfilled our criteria for severe anxiety symptoms also have significantly higher anxiety scores in all three areas of everyday-life. On the whole, the correlations with potential influencing factors are very similar, indicating that especially persons with low socioeconomic status are more strongly stressed by both everyday-life and clinically significant anxieties. Also, the equally robust correlations concerning self-evaluated health show a clear overlap, underlining the assumption of a continuum of anxiety. Although our data cannot provide conclusive evidence, the present results suggest that everyday-life anxieties and clinical anxieties may be present in an intensity continuum which then, at the clinical end, differentiates into various partial dimensions that are definable by their contents (c.f., for example Endler and Kocovski, 2001).

## Conclusion

The present study successfully tested an efficient and economical survey instrument for clinical and everyday-life anxieties in the general population. The compact screening instrument will allow an easier investigation of time series and possible correlations with (macro-) social factors. Beyond this first objective, our study provides new insights into the intensity of everyday-life anxieties in the general population, their correlations with clinical anxieties, potential influencing factors on clinical and everyday-life anxieties and, for the first time, the course of the development of anxiety during different stages of life. Our estimates of the prevalence of severe anxiety symptoms correspond well with prevalence rates of anxiety disorders found in previous epidemiological studies. Altogether, it is apparent that the majority of the general population is only slightly stressed by clinical and everyday-life anxieties. At the same time, however, a substantial minority of the population is strongly affected by anxiety. The results are well compatible with a dimensional understanding of anxiety. This view is further supported by the finding that the observed influencing factors were the same for everyday-life anxieties and clinical anxieties. These factors also include rarely investigated possible protective factors, such as intellectual activity.

However, despite the striking similarities between clinical and everyday-life anxieties, some of the correlations with the various factors (e.g., age, type of media use) are quite heterogeneous. The individual dimensions of everyday-life anxieties or types of anxiety disorders can also have opposing effects, so that a differentiated view is essential. The observed effects are rather small, however, plausible. Although the correlative design of the study does not allow causal statements, our results pose many interesting starting points for future experimental intervention studies. Together with time series from repeated representative surveys, we expect the present findings to provide a better understanding of the processes that underlie everyday-life and clinical anxieties.

## Author contributions

JM, SS study concept. JM, SS, and DA study design and manuscript preparation. DA data analysis.

### Conflict of interest statement

The authors declare that the research was conducted in the absence of any commercial or financial relationships that could be construed as a potential conflict of interest.

## Footnotes

^1^The instrument can be obtained from the authors on request.

^2^The questionnaire on leisure activities was formulated in close relation to previously published research. The frequencies of mental activities, social contacts, and physical activities/sports were assessed according to previous published research indicating that these life-style behaviors are protective factors in mental health (see Velten et al., 2014). The frequencies of computer gaming and time spent on the Internet were assessed according to previous research showing that both are related to a higher risk for mental illness (frequency of computer gaming, Mentzoni et al., 2011; time spent on the Internet Yau et al., 2013).
